# Supplementary material for: Content-rich biological network constructed by mining PubMed abstracts
Source: BMC Bioinformatics. 2004 Oct 8;5:147. doi: 10.1186/1471-2105-5-147 (PMC528731; doi:10.1186/1471-2105-5-147)
Supplement: Additional File 2 — The original results of the above study (non-essential files are deleted to keep the file size under the limit set by BMC bioinformatics). [file 1471-2105-5-147-S2.bz2 › chilibotAdditionalFile2/dip10/10ID9009191E31/html/ACTN2_CALM1.html]

 


 **ACTN2** and **CALM1** 
  
Found 8 abstracts in PubMed, retrieved 8.  
 

 What does Google say? 
 PDF only 
| .edu only 

---

**Interactive relationship** (e.g. stimulation, inhibition, etc)

**Inhibitory relationship**- Furthermore, this inactivating effect of calmodulin  [ **CALM1** ]  can be prevented by coexpressing a region of the cytoskeletal protein alpha actinin2  [ **ACTN2** ]  known to interact with the CO region of NR1.  Ref: 9728925 Neuron, 1998
**Neutral relationship**

**Non-interactive relationship** (e.g. studied together, co-existance, homology, etc.)

- Like Ca calmodulin  [ **CALM1** ] , autophosphorylated CaMKII competes with alpha actinin 2  [ **ACTN2** ]  for binding to NR1.  Ref: 12379661 J Biol Chem, 2002
- NMDA receptor activity is also regulated by the intracellular calcium concentration through activation of various calcium dependent proteins, including calmodulin  [ **CALM1** ] , calcineurin, protein kinase C, and alpha actinin 2  [ **ACTN2** ] .  Ref: 12388592 J Neurosci, 2002
